# Spatiotemporal Anomaly Detection in Distributed Acoustic Sensing Using a GraphDiffusion Model

**DOI:** 10.3390/s25165157

**Published:** 2025-08-19

**Authors:** Seunghun Jeong, Huioon Kim, Young Ho Kim, Chang-Soo Park, Hyoyoung Jung, Hong Kook Kim

**Affiliations:** 1 Department of AI Convergence, Gwangju Institute of Science and Technology, Gwangju 61005, Republic of Korea; zldzmfoq12@gm.gist.ac.kr; 2 Korea Photonic Technology Institute, Gwangju 61007, Republic of Korea; pcandme@kopti.re.kr (H.K.); kimyh@kopti.re.kr (Y.H.K.); 3 Department of Electrical Engineering and Computer Science, Gwangju Institute of Science and Technology, Gwangju 61005, Republic of Korea; csp@gist.ac.kr

**Keywords:** distributed acoustic sensing, graph neural network, diffusion model, anomaly detection, generative model, spatial–temporal modeling

## Abstract

Distributed acoustic sensing (DAS), which can provide dense spatial and temporal measurements using optical fibers, is quickly becoming critical for large-scale infrastructure monitoring. However, anomaly detection in DAS data is still challenging owing to the spatial correlations between sensing channels and nonlinear temporal dynamics. Recent approaches often disregard the explicit sensor layout and instead handle DAS data as two-dimensional images or flattened sequences, eliminating the spatial topology. This work proposes GraphDiffusion, a novel generative anomaly-detection model that combines a conditional denoising diffusion probabilistic model (DDPM) and a graph neural network (GNN) to overcome these limitations. By treating each channel as a graph node and building edges based on Euclidean proximity, the GNN explicitly models the spatial arrangement of DAS sensors, allowing the network to capture local interchannel dependencies. The conditional DDPM uses iterative denoising to model the temporal dynamics of standard signals, enabling the system to detect deviations without the need for anomalies. The performance evaluations based on real-world DAS datasets reveal that GraphDiffusion achieves 98.2% and 98.0% based on the area under the curve (AUC) of the F1-score at *K* different levels (F1*_K_*-AUC), an AUC of receiver operating characteristic (ROC) at *K* different levels (ROC*_K_*-AUC), outperforming other comparative models.

## 1. Introduction

Distributed acoustic sensing (DAS) technology senses infrastructure, which transforms standard optical fiber cables into dense distributions of acoustic sensor arrays [1]. Further, DAS can monitor dynamic events for long distances using backscattered illumination in fiber-optic cables. The applicability of DAS across various domains, such as seismic activity detection [2], CO_2_ storage monitoring [3], ship trajectory tracking [4], oceanographic observation [5], and pipeline safety monitoring [6,7], via vibration-based pattern recognition, is well-documented.

As DAS data become more available, deep learning approaches have increasingly been adopted for classifying events and detecting anomalies. The one-class support vector machine (OCSVM) [5] and convolutional neural network (CNN)-based autoencoder methods [8,9] demonstrate remarkable performance in this task. Transfer learning [10], few-shot learning [11], and zero-shot learning [12] approaches have been employed to address the lack of generalizability due to unseen types of events and limited quantities of label data.

Despite these advancements, DAS-based anomaly detection suffers from some critical problems. First, most models treat the DAS signals in one-dimensional (1D) flat form or 2D spectrogram-like formats, but this treatment leads to the loss of the spatial structure of the DAS system, where each channel is mapped to a sensor along the optical fiber. The representations ignore the interchannel relationships, which are crucial for the detection of spatially distributed events.

Second, CNN-based temporal models, including combinations of multiscale CNNs with hidden Markov models [13] or CNNs with recurrent neural networks (RNNs) [14], exhibit limitations in anomaly detection with DAS signals. The CNN relies on fixed and translation-invariant filters under the stationarity assumption [15] and is poorly suited to dynamic spectral shifts or propagating vibrations. Although essentially sequential, RNNs suffer from vanishing gradients and short memory, inhibiting their management of long-range and nonlinear dependencies common in spatially distributed acoustic signals [16].

These limitations require the adoption of more expressive and robust generative models to cope better with complex temporal patterns in DAS data. Furthermore, these limitations highlight the need for a more structured method to consider the spatial layout of the DAS sensor arrays. This paper proposes a novel method, GraphDiffusion, the integration of a graph neural network (GNN), and the conditional denoising diffusion probabilistic model (DDPM), to address the spatial and temporal modeling challenges in DAS-based anomaly detection.

Recently, GNNs have performed anomaly detection well [17]. The GNN, notably the graph convolutional network (GCN) [18,19,20], is suitable for representing the spatial topology of DAS systems, where the channels map to physical locations along the fiber. The fiber layout of the DAS system is explicitly represented as a graph, where channels are modeled as nodes and edges are determined by physical proximity. In installations (e.g., perimeter fences) where the optical fibers are looped along the bottom and top, this would allow the connection of the neighbors, including diagonally or symmetrically aligned ones, to respond together to the same events. This approach embeds 2D coordinates for each channel and forms edges based on the Euclidean distance, including horizontal, vertical, and diagonal connections. Edge weights are calculated as the inverse of the distance, enabling the model to focus on spatially relevant dependencies. Graph building facilitates GCNs to discover local and distributed vibrational patterns that are crucial for spatially extending or propagating anomaly detection.

Although the GNN component extracts spatial correlations within the DAS array, the conditional DDPM offers a mechanism to model the temporal progression of signals. The conditional DDPM discovers the distribution of normal temporal patterns by learning an iterative denoising process, which is trained on a generative task by incrementally corrupting clean signals and learning to invert noise [21]. During inference, reconstruction after denoising serves as the baseline for comparison, and anomalies can be detected via significant reconstruction errors. The probabilistic and generative properties of DDPMs facilitate the handling of nonstationary and nonlinear dynamics, even those with low signal-to-noise ratios. Anomaly detection via diffusion-based models has performed well in diverse fields (e.g., medical and industrial monitoring) because it detects subtle irregularities and remains robust even with noisy or complex data [21,22,23].

GraphDiffusion addresses the limitations of past approaches by modeling the spatial dependence and temporal dynamics simultaneously. GraphDiffusion obtains the spatial context from the DAS layout via the GNN and employs it in the denoising process as a spatial condition of the conditional DDPM. The temporal denoising procedure can consider the spatial signal patterns observed throughout the DAS array by employing these embeddings as conditions for the DDPM. Experiments on DAS datasets are performed and compared with other models to demonstrate the performance of the proposed GraphDiffusion method by using the metrics, such as the area under the curve (AUC) of F1-score at *K* different levels (F1*_K_*-AUC) and the AUC of receiver operating characteristic (ROC) at *K* different levels (ROC*_K_*-AUC).

The remainder of this paper is organized as follows. Section 2 presents a brief review of the anomaly-detection method applied to DAS and multivariate time series (MTS). Section 3 explains the fundamental principles of DAS, and Section 4 describes the site configuration, DAS system parameters, and data-collection method. Then, Section 5 proposes the GraphDiffusion, comprising the GNN and conditional DDPM, and Section 6 outlines the dataset, implementation details, and evaluation protocol used to validate the anomaly-detection performance of GraphDiffusion. Finally, Section 7 presents the quantitative results and ablation studies confirming the effectiveness of GraphDiffusion, and Section 8 concludes the paper.

## 2. Related Work

The DAS and anomaly detection based on MTS methods have evolved from traditional rule-based methods to deep learning techniques that better capture complex patterns. Convolutional, recurrent, transformer, diffusion, and graph-based models have all contributed to valuable advances. However, many still ignore the spatial relationships between vital sensor channels in DAS. This section reviews these approaches and highlights the necessity of methods that jointly consider spatial and temporal dependencies.

### 2.1. Anomaly Detection in Distributed Acoustic Sensing

The DAS method enables the high-definition monitoring of vibrations along standard optical fiber and is employed in seismic activity tracking [2], ship motion monitoring [4], and cable monitoring [5,6,7]. Traditional signal enhancement schemes, such as empirical mode decomposition [24,25], were employed to enhance signal-to-noise levels for anomaly detection. Furthermore, a threshold-based statistical model was used to detect vibration in DAS [26]. However, these rule-based approaches are vulnerable and cannot generalize to various DAS environments.

With the development of deep learning, CNNs and RNNs have been employed to handle anomaly detection [8,9,10,14]. In [13], an end-to-end model combining a multiscale CNN and a hidden Markov model was proposed, where the CNN learns local structural features and the hidden Markov model learns sequential information to improve classification performance. An unsupervised deep clustering method for anomaly detection in phase-sensitive optical time domain reflectometry (Φ-OTDR) traces was proposed [9]. This approach perfectly suppresses noise and identifies outliers in unlabeled settings, demonstrating advantages in practicality.

Nonetheless, most previous models treat DAS observations as simple time series or spectrograms, neglecting the spatial structure of the fiber channel. This abstraction hinders the detection of spatially distributed or propagating anomalies.

### 2.2. Anomaly Detection in Multivariate Time Series

MTS data have been applied in industrial monitoring, medical, and finance fields, which require correlated distributed data analysis. Research on anomaly detection using this approach has been conducted. Existing methods based on statistical modeling and autoencoders have been studied, and the variational autoencoder [27] and its extended method, Donut, effectively detect anomalies by reconstructing normal patterns in MTS. However, these models are often over-smoothed and have limited expressiveness, especially in high-dimensional or strongly correlated data.

Recently, the transformer-based architecture has been intensively studied regarding anomaly detection in MTS because it can represent complex variable interactions and long-range temporal dependencies. Anomaly Transformer [28], TranAD [29], iTransformer [30], and Mamba adaptive anomaly transformer (MAAT) [31] have gradually improved anomaly detection performance by advancing attention mechanisms and architectures. Despite these improvements, the transformer-based architecture generally does not consider the spatial proximity between sensors, which is important in structured environments, such as those using DAS.

In addition, diffusion-based generative models capable of capturing complex temporal dynamics have been employed as an architecture for robust reconstruction-based anomaly detection in MTS. Various models have demonstrated scalable and high-quality reconstruction by introducing a diffusion mechanism [21]. However, most diffusion models ignore the spatial dependency between channels, limiting their application in scenarios where sensor placement forms crucial correlations.

### 2.3. Anomaly Detection Using Graph Neural Networks

The GNN is a highly effective tool for learning structured data in non-Euclidean domains, enabling it to propagate information across irregular graph topologies [18,19,20]. Unlike conventional convolutional or recurrent models, which are suitable for grid-structured data, the GNN captures dependencies between nodes, making it suitable for distributed sensing applications [32,33,34,35,36]. By representing DAS channels with connected graph nodes based on physical proximity, the GNN can more accurately model relationships between channels, supporting anomaly detection.

Recently, research has employed GNN-based frameworks for MTS anomaly detection to capture complex interactions between sensors. These methods highlight the limitations of processing sensor data independently by modeling spatial or functional relationships, taking advantage of the graph structure. These studies have demonstrated that combining GNNs with temporal modeling methods, such as the variational autoencoder, attention mechanisms, or masking strategies, can more successfully detect anomalies [32,33,34,35,36]. Combining temporal modeling with sensor relationship-learning significantly improves anomaly detection performance in various applications.

Unlike models that handle a time series as independent signals or 2D patterns, GNNs offer an effective method of capturing spatial arrangements and relationships between sensors. The GNN can detect complex and dispersed anomalies that temporal or spectral models frequently miss by creating graphs that represent connections. Nevertheless, despite these benefits, the GNN has not yet been widely applied for DAS anomaly detection. The proposed work aims to bridge this gap using the spatial structure learning capabilities of the GNN as a component of a spatiotemporal anomaly-detection system. Furthermore, this study proposes combining the conditional DDPM with the GNN to employ the temporal modeling of conditional DDPM and the spatial structure encoding capabilities of GNNs.

## 3. Principles of the DAS System

Using standard optical fiber, DAS detects acoustic disturbances and external vibrations in real time with high resolution. The DAS system can localize and characterize events with fine spatial and temporal granularity by measuring phase changes in Rayleigh backscattered signals along the fiber. This section describes the fundamental operating principles of DAS, including critical system parameters that support robust sensing capabilities for security and infrastructure monitoring, the trade-off of pulse width selection, and the physical interpretation of phase measurements.

### 3.1. Fundamental DAS Principles

The DAS system functions according to the principle of Φ-OTDR. This system involves launching a narrow-linewidth laser pulse into an optical fiber and continuously monitoring the Rayleigh backscattered light that returns from each location along the fiber. The phase and intensity of the backscattered signal are modulated when an external acoustic or vibrational disturbance is applied to the fiber, causing a local strain or deformation. Analyzing these backscattered signals in the time domain permits detecting and localizing external events along the entire length of the fiber in real time.

### 3.2. System Parameters and Mathematical Relationships

The performance characteristics of DAS systems are governed by several parameters that determine the spatial and temporal capabilities of the system. The spatial sampling distance Δz along the fiber is fundamentally determined by the sampling rate of the data-acquisition system and is expressed as follows:

(1) $$\Delta z=\frac{c_{0}\cdot t_{s}}{2n}$$

where $c_{0}$ denotes the speed of light in a vacuum, $t_{s}$ represents the sampling interval, and *n* indicates the refractive index of the optical fiber core (typically about 1.468 for standard single-mode fiber). The factor of 2 accounts for the round-trip propagation of light in the fiber. The temporal sampling distance, Δt, is directly related to the pulse repetition rate $f_{p}=1/\Delta t$ of the interrogating laser.

However, the spatial resolution of the system is primarily determined by the gauge length $L_{g}$, corresponding to the physical length of fiber over which the acoustic signal is integrated. The gauge length is intrinsically linked to the pulse width τ of the interrogating laser pulse:

(2) $$L_{g}=\frac{c\cdot \tau }{2}$$

where *c* denotes the speed of light in optical fiber (typically $c=c_{0}/n\approx 3\times 10^{8}/1.468\approx 2.04\times 10^{8}$).

### 3.3. Pulse-Width Trade-Offs

This relationship establishes a fundamental trade-off between spatial resolution and system sensitivity. Shorter pulse widths result in improved spatial resolution by reducing the gauge length, enabling more precise localization of acoustic events. However, because the shorter gauge length integrates acoustic signals over a smaller fiber section, potentially lowering the signal-to-noise ratio, this improvement comes at the cost of decreased sensitivity. On the other hand, longer pulse widths increase the gauge length, improving sensitivity through signal integration across a larger fiber segment but lowering spatial resolution.

The application requirements must be carefully considered when choosing the ideal pulse width, striking a balance between the need for accurate event localization and sufficient detection sensitivity. For most security and monitoring applications, typical DAS systems use pulse widths between 10 and 100 ns, which correspond to gauge lengths of roughly 1 to 10 m. This offers a workable compromise between resolution and sensitivity.

### 3.4. Phase Measurement and Physical Interpretation

The phase (*φ*), which is measured in radians and represents the optical path difference caused by external disturbances like vibration waves and acoustic sound pressure, is the basic unit of measurement in DAS systems. Important physical details about the optical fiber’s mechanical deformation and acoustic field are contained in this phase measurement. The relationship between phase change and fiber strain is governed by the photo-elastic effect, and phase change is expressed as follows:

(3) $$\Delta \varphi =\frac{4\pi n}{\lambda_{l}}L_{g}\varepsilon_{x,t}$$

where $\lambda_{l}$ represents the interrogating laser wavelength, *n* indicates the refractive index of the optical fiber core, and $\varepsilon_{x,t}$ represents the longitudinal strain along the fiber at spatial position *x* and time *t*. The gauge length $L_{g}$ is computed using Equation (2). The detection of mechanical deformations as small as one nano-strain (1 × 10^−9^) is made possible by this relationship, which shows that phase measurements directly correlate with nano-strain levels.

The relationship between the fiber strain and the applied acoustic field is what makes the conversion from phase measurements to acoustic pressure possible. When acoustic waves hit fiber-optic cables, the strain that is caused is linked to the acoustic pressure through the mechanical properties of the cable and how well it couples. The acoustic pressure *P* can be derived from the measured phase as follows:

(4) $$P=\zeta \varepsilon_{x,t}\;$$

where ζ represents the elastic coefficient of the fiber, and $\varepsilon_{x,t}$ denotes the longitudinal strain as described in Equation (3). This dual relationship among the phase, strain, and acoustic pressure lets DAS systems work as both mechanical strain sensors and acoustic pressure detectors. Because of this connection, these systems can be used for a wide range of purposes, such as monitoring the health of structures, detecting earthquakes, and acoustic surveillance.

## 4. Data Collection

This work established a large DAS-based experimental setup to simulate real-world security threats and gather data to test the proposed anomaly-detection framework. This section explains how to set up a site, the DAS system settings, and how to collect data. To improve detection coverage, a dedicated test environment was set up using a dual-height fiber-optic installation strategy. Different physical activities were then performed in a planned way to create representative datasets.

### 4.1. Site Configuration and Installation

**Figure 1:**
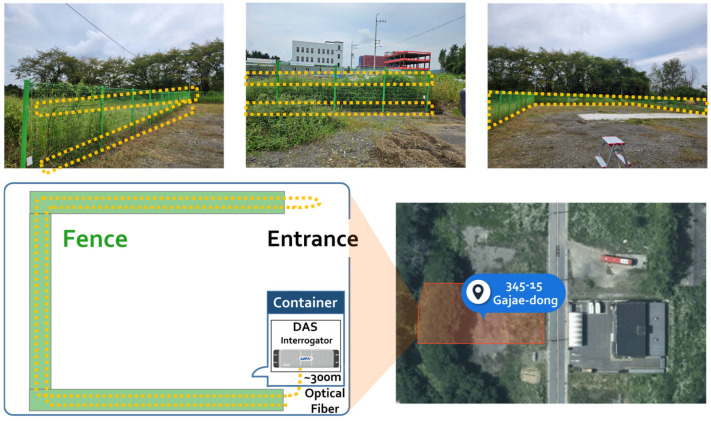
Illustrations of an experimental environment (testbed) where a fiber cable is attached to the fence perimeter (345-15 Gajaeo-dong, Pyeongtaek, Gyeonggi-do, Republic of Korea).

The experimental deployment was conducted at a designated test site with a perimeter security fence system integrated with DAS technology. Figure 1 depicts the experiment environment, optical fiber, and DAS interrogator installation. The security fence was constructed using a U-shaped configuration about 2 m in height, offering a controlled environment for intrusion-detection testing. The optical fiber cable was carefully put along the perimeter of the fence to make a distributed sensing network that could detect and localize attempts at intrusion. The DAS interrogator unit was kept in a weatherproof container at the entrance to the site. This kept the sensitive optical equipment safe while still allowing for easy access for system monitoring and maintenance.

A standard single-mode telecommunications fiber with improved mechanical protection that is appropriate for outdoor installation makes up the fiber-optic cable used in this study. To improve detection reliability and offer redundant sensing coverage, a dual-fiber installation strategy was used; 310 sensing channels are supported throughout the installation. Two different height levels were used for the installation of the optical fibers: 30 cm above the ground and 160 cm above the ground. A continuous sensing loop that covers both lower and upper fence sections is created by the fiber cable ascending along one side of the fence posts to the upper mounting point and then descending along the opposite side. This dual-height configuration was designed using a round-trip topology.

Cable ties were employed to securely attach the cables to the fence structure, making sure that the fiber and fence framework were in contact with each other to maximize vibration transmission. This round-trip installation method gives the system better coverage of space and allows it to compare the two height levels, which makes it better at distinguishing the difference between different types of intrusion attempts, like climbing and cutting. The total monitored perimeter length was about 300 m, and each fiber strand covered the entire distance, which doubled the sensing density along the fence line.

### 4.2. DAS Operational Parameters

The phase-sensitive DAS unit used in this study was produced by the Korea Photonics Technology Institute. Table 1 summarizes the operational parameters, where Msps denotes mega samples per second. The parameters were chosen to optimize the detection performance for perimeter security applications, providing sufficient spatial resolution for event localization while maintaining sufficient temporal resolution for prompt detection.

### 4.3. Scenario Design and Data Acquisition

To simulate realistic security threats, a variety of physical activities were methodically carried out during several sessions from 2023 to 2024. The dataset used in this paper was gathered between 4 September and 6 September 2023. To evaluate the DAS system’s detection sensitivity and pattern discriminability, the experiments were made to produce unique vibrational and acoustic signatures. The dataset contains recordings of human movement, digging, ladder intrusions, and fence vibration.

To simulate physical tampering, the fence was deliberately shaken and struck to produce a fence vibration scenario. Table 2 describes each event scenario and the data associated with the scenario during data collection. In the digging scenario, digging actions were performed using a shovel near the fence line. Ladder intrusion events were simulated using a ladder to climb up the fence and capture signals related to ladder placement, elevation, and descent. Human movement activities included walking and running experiments conducted under various conditions, and sensitivity tests were performed at distances of between 1 and 5 m around the fence to evaluate detection performance under various proximity conditions.

In this study, “walking” and “running” are defined as normal events, whereas “fence impact,” “fence shaking,” “digging,” and “ladder intrusions” were treated as anomalies. From the normal portion of the data, 398,900 traces were employed as the training dataset, and the testing and validation datasets comprise 22,300 normal traces and 24,000 anomalous traces, ensuring a balanced and realistic performance evaluation. The DAS dataset is represented as an MTS $X\in R^{T\times C}$, where *T* denotes the number of traces and *C* = 310 is the number of distributed sensing channels.

## 5. GraphDiffusion Methods

**Figure 2:**
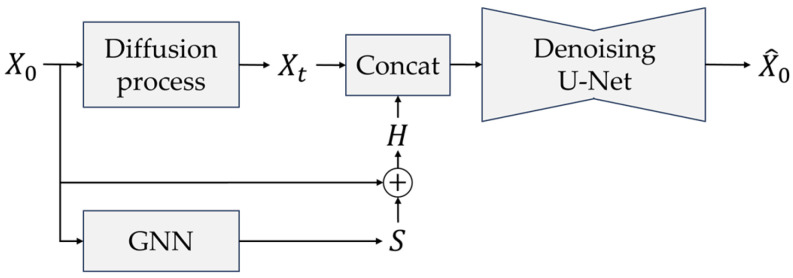
Structure of the proposed GraphDiffusion model.

GraphDiffusion combines a GNN with a conditional DDPM to jointly learn spatial and temporal patterns in DAS signals. Figure 2 shows the overall structure of the proposed GraphDiffusion. In Figure 2, the GNN extracts a spatial embedding *H* among DAS channels, encoding how vibrations propagate across the fiber layout. In parallel, a diffusion process perturbs the input $X_{0}$ with Gaussian noise to learn the temporal distribution of normal signals. The spatial embeddings from the GNN and the temporally corrupted signals $X_{t}$ are concatenated and passed into a denoising U-Net, which progressively reconstructs a clean signal, ${\hat{X}}_{0}$. The hierarchical structure of U-Net allows it to combine local details with the global context, while conditioning it on the spatial information from the GNN. This architecture enables GraphDiffusion to capture complex spatiotemporal dependencies for generative anomaly detection in DAS systems.

### 5.1. DAS Signal Preprocessing

For raw DAS data $X\in R^{T\times C}$, *T* denotes the number of traces, and *C* is the number of channels (sensors) along the fiber. With a stride *S*, these sequences are divided into windows of a fixed length of size *W*. The diversity of training samples is increased using a sliding window technique with *S* < *W* to create overlapping segments during training. A nonoverlapping windowing strategy is employed during validation and testing by setting the stride equal to the window size (i.e., *S* = *W*) to ensure a consistent and nonredundant evaluation protocol. Thus, each windowed segment creates an input tensor $X_{0}\in R^{W\times C}$.

### 5.2. GNN-Based Spatial Representation Learning

**Figure 3:**
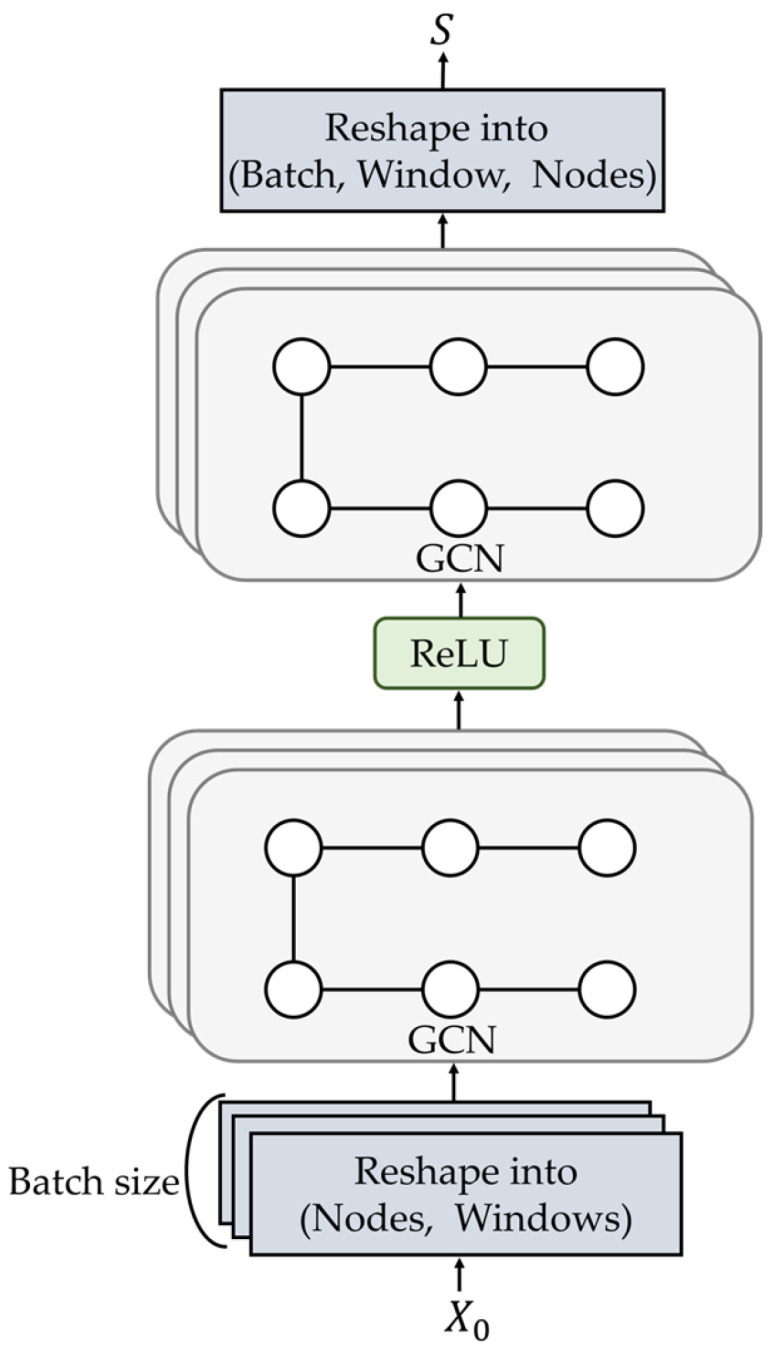
Structure of the graph neural network in the proposed GraphDiffusion model.

This section details the graph-construction process that encodes the physical topology of fiber-optic placement sensors and the design of GNN that are employed in the proposed GraphDiffusion to model the spatial structure of DAS signals. Figure 3 illustrates the structure of GNN, where windowed segment $X_{0}$ is processed by a two-layer GCN with the rectified linear unit (ReLU) activation function [37] applied between layers. The resulting output is spatial feature map *S*. These components allow the proposed work to capture neighborhood dependencies and spatial correlations that are essential for robust anomaly detection.

#### 5.2.1. Graph Construction

Each window of DAS data is converted into an undirected graph *G* = (*V*, *E*) where each node $v_{i}\in V$ represents a DAS sensor, to model the spatial dependencies in the DAS system. The graph structure was explicitly derived from the actual dual-fiber installation described in Section 4.1, where two parallel optical fibers were installed at different heights along the perimeter fence.

**Figure 4:**
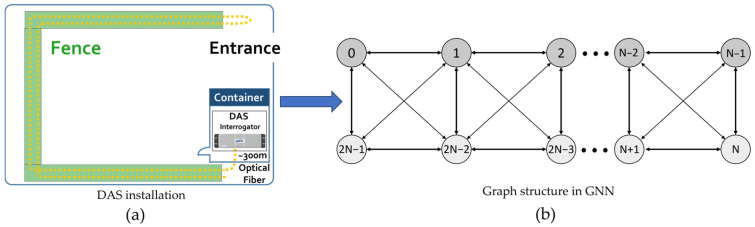
Illustrations of (**a**) the installation of DAS sensors and (**b**) their corresponding graph topology.

Figure 4 depicts the graph structure as a two-row topology to imitate the installation of DAS, enabling spatial dependency modeling, including horizontal, vertical, and diagonal signal propagation. Each sensor is assigned a virtual 2D coordinate to represent the two-row topology. As shown in Figure 4a, the DAS sensors were deployed in a dual-fiber configuration along the bottom and top of the fence. As shown in Figure 4b, the top-half channels, indexed from 0 to *N* − 1, are assigned uniformly spaced horizontal positions at a vertical level of *y* = 0 to mimic the upper cable run. The bottom-half channels, indexed from *N* to 2*N* − 1, are assigned to *y* = 1 in a horizontally mirrored order to replicate the inverted orientation of the lower cable run. This configuration is horizontally mirrored but vertically aligned. Every channel has a coordinate $\left(x_{i},y_{i}\right)$, where $y_{i}\in \left\{0,1\right\}$ indicates the vertical position and $x_{i}$ indicates the horizontal position. An adjacency matrix with a fixed spatial distance threshold *d* is created to specify the graph topology. Edges in this adjacency matrix are created between any pair of nodes (*i, j*) if their Euclidean distance is less than or equal to *d*:

(5) $$dist\left(i,j\right)=\sqrt{{\left(x_{i}-x_{j}\right)}^{2}+{\left(y_{i}-y_{j}\right)}^{2}}\leq d$$

Inversely proportional to the Euclidean distance, each edge is assigned a weight ($w_{ij}=1/\left(dist\left(i,j\right)+\delta \right)$, where *δ* is a small constant to prevent division by zero. The resulting adjacency matrix $A\in R^{C\times C}$ encodes the physical geometry of DAS channels, enabling the GCN layers to learn symmetric and local spatial patterns efficiently, including distributed or subtle propagations. Self-loops are added to ensure that each node’s unique features are included during message passing and to preserve per-channel information. This structure is implemented as a [2,E] matrix that specifies the source–target edge pairs and is encoded using a static edge index that is constant across batches.

#### 5.2.2. GNN Architecture in the Proposed GraphDiffusion

A two-layer GCN is employed for graph-based spatial feature extraction [19]. In the first layer, the input $X_{0}$ is passed through a GCN followed by an ReLU activation function [37], projecting them into a hidden representation. The GCN applies a convolution over the shared graph structure for every batch sample, reshaping the features to fit the initial input dimensions. Formally, the spatial feature map $S\in R^{W\times C}$ is computed as follows:

(6) $$S=GCN\left(ReLU\left(GCN\left(X_{0}\right)\right)\right)$$

The GCN output S is combined with the original input $X_{0}$ via an elementwise sum operation to create the final spatial embedding H:

(7) $$H=S+X_{0}$$

Interchannel dependencies resulting from the actual sensor arrangement and detected signal correlations are encoded in these spatial embeddings. The temporal denoising procedure can consider spatial signal patterns observed throughout the DAS segment using these embeddings as conditions for the conditional DDPM.

### 5.3. Diffusion-Based Temporal Modeling

We used a conditional DDPM [38] that incorporates a U-Net-based denoising network $\epsilon_{\theta }$ [39] to model the temporal structure of DAS signals. The spatial context obtained from the DAS layout can be employed to guide the denoising process due to this conditional design. We applied graph-based spatial embeddings produced by the GNN to condition the diffusion model. This module design can simultaneously encode high-level contextual information across the signal and capture local temporal dependencies [21,40,41].

#### 5.3.1. Denoising U-Net Architecture

**Figure 5:**
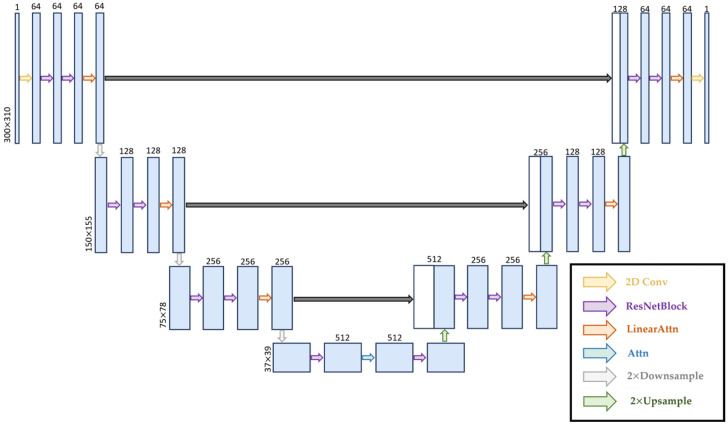
Network architecture of denoising U-Net used in the proposed GraphDiffusion model.

The stepwise denoising procedure, which is essential for DDPMs, is learned using U-Net. Figure 5 illustrates the architecture of U-Net, which applies a hierarchical structure of downsampling and upsampling layers with feature multipliers of 1, 2, and 4, enhanced with residual blocks and attention mechanisms, to operate on noisy input and recover the original clean signal. Reshape-based size-aware convolutional layers are applied to implement downsampling and upsampling operations to guarantee compatibility with nonsquare DAS inputs $X_{0}$. Features from matching downsampling blocks are concatenated in the upsampling path using skip connections. A two-layer multilayer perceptron, for which output modulates the residual blocks via featurewise linear modulation-style scale and shift parameters, is employed to achieve timestep conditioning after sinusoidal positional embedding. Additionally, the model allows for self-conditioning, promoting improved temporal consistency in predictions by concatenating a previously denoised output to the input during training.

#### 5.3.2. Diffusion Process

Gaussian noise is added to a clean window $X_{0}\in R^{W\times C}$ over t time steps during training via the diffusion process:

(8) $$X_{t}=\sqrt{{\bar{\alpha }}_{t}}X_{0}+\sqrt{1-{\bar{\alpha }}_{t}}\epsilon ,\;\epsilon \sim N\left(0,I\right)$$

where ${\bar{a}}_{t}$ represents the cumulative product of noise schedule coefficients, defined as ${\bar{\alpha }}_{t}=\prod_{s=1}^{t}\alpha_{s}$, where $\alpha_{s}=1-\beta_{s}$, and $\beta_{s}\in \left(0,1\right)$ controls the noise level at each timestep. Intuitively, ${\bar{\alpha }}_{t}$ indicates the proportion of the original signal $X_{0}$ preserved at step t, and $1-{\bar{\alpha }}_{t}$ reflects the amount of added noise. As t increases, ${\bar{\alpha }}_{t}$ decreases, gradually replacing the signal with pure noise.

Given the noisy input $X_{t}$ in Equation (8), the diffusion timestep t, and the GNN-derived condition H in Equation (7), the model is trained to reverse this process and predict the added noise ϵ while minimizing the following loss:

(9) $$L_{dif}=E_{X_{0},t,\epsilon }\left[{\left\|\epsilon -\epsilon_{\theta }(X_{t},t,H)\right\|}^{2}\right]$$

where $\epsilon_{\theta }$ denotes the denoising network.

At inference, we added noise to an input $X_{0}\to X_{t}$ and then denoised the input back to $X_{t}\to {\hat{X}}_{0}$. We iteratively denoised the previous input t times, starting from ${\hat{X}}_{t}$ = $X_{t}$. The denoised ${\hat{X}}_{n-1}$ is calculated as follows:

(10) $${\hat{X}}_{n-1}=\frac{1}{\sqrt{{\bar{\alpha }}_{n}}}\left({\hat{X}}_{n}-\frac{\beta_{n}}{\sqrt{1-{\bar{\alpha }}_{n}}}\epsilon_{\theta }\left({\hat{X}}_{n},n,H\right)\right)+{\hat{\beta }}_{n}z,n=t,t-1,\ldots ,1$$

where ${\hat{\beta }}_{n}=\frac{1-{\bar{\alpha }}_{n-1}}{1-{\bar{\alpha }}_{n}}\beta_{n}\;(\beta_{n}\in \left(0,1\right))$, and $z∼N\left(0,I\right)$ if *n* > 1, otherwise *z* = 0. The anomaly score at trace *w* is calculated using the reconstruction error between the original input signal $X_{0}=\left[e_{0},e_{1},\ldots ,e_{W}\right]$ and denoised signal ${\hat{X}}_{0}$ = [${\hat{e}}_{0},{\hat{e}}_{1},\ldots ,{\hat{e}}_{W}$] where each trace element $e_{w}$ and ${\hat{e}}_{w}\in R^{C}$ is a feature vector:

(11) $$s_{w}={\left\|e_{w}-{\hat{e}}_{w}\right\|}_{2\;\;\;}^{2}$$

This anomaly score is used for verifying the anomaly-detection models with evaluation metrics in Section 6.4.

This design allows the model to learn fine-grained temporal patterns and global contextual dependencies via the resolution hierarchy of U-Net. By conditioning the denoising process on encoded temporal representations, the model can better generalize to complex and subtle anomalies in DAS data.

## 6. Experimental Setup

This section presents a setup of experiments designed using a proprietary DAS dataset collected in realistic infrastructure monitoring scenarios to verify the effectiveness of the GraphDiffusion method. The purpose of the experiments is to assess the model’s capacity to identify subtle, spatially correlated anomalies. This section describes the details, implementation settings, and evaluation metrics of the dataset to ensure reproducibility and a fair comparison with comparative models.

### 6.1. Dataset

A proprietary DAS dataset for anomaly detection in MTS is employed for the experiments. A time series of signals with *C* = 310 channels (i.e., fiber-sensing channels) comprises the dataset, with $T_{train}$ = 398,900 traces in the training set and $T_{val}\;$= $T_{test}\;$= 46,300 traces in each of the validation and test sets. Note that there were no overlaps among the training, validation, and test sets.

Let $X=\left[e_{1},e_{2},\ldots ,e_{T}\right]\in R^{T\times C}$ represent an MTS with *C* dimensions and *T* traces to formalize the data. A set of windowed sequences is created by dividing each dataset into overlapping windows of length *W* = 300 using a stride of *S* = 150. Nonoverlapping windows are employed for the test and validation sets, and the majority vote of the included traces determines each window label. The 24,000 anomalous and 22,300 normal traces in each set allow a balanced and realistic performance evaluation.

### 6.2. Implementation Details

The GNN in GraphDiffusion has a hidden-layer size that is half of the window size. A linear noise schedule increases from $\beta_{1}=10^{-4}$ to $\beta_{T}=0.02$, with a diffusion step count of 100. Only normal DAS data are used for training, and the Adam optimizer is employed with a batch size of 32 and a learning rate of 1 × 10^−3^. A learning rate scheduler is used, which increases the learning rate linearly for the first 10% of training steps and then decreases it linearly afterward. All experiments were implemented using three Nvidia A5000 GPUs (NVIDIA Corporation, Santa Clara, CA, USA) and PyTorch version 1.13.1. The average training time per epoch of the proposed model is 204 s, and the experiments were conducted over 40 epochs. At inference, the model generates 300 traces in 0.0004 s (≈1.3 µs per trace).

### 6.3. Comparative Models

This work compares the proposed model to a set of comparative models to assess its effectiveness. The pipelines from previous anomaly detection in DAS studies were reimplemented, including an anomaly-detection model using OC-SVM [41] and a generative learning model with an autoencoder [9].

Furthermore, the comparative models were six recent generative models: the AnomalyTransformer [28], TranAD [29], iTransformer [30], MAAT [31], DiffusionAE [21], and graph deviation network (GDN) [32]. For anomaly scoring, TranAD uses a two-stage transformer architecture that combines reconstruction and prediction goals. AnomalyTransformer measures association discrepancies in temporal attention to quantify anomaly likelihoods. For long-term series, the iTransformer uses an inductive bias and a shifted windowing mechanism to improve scalability and efficiency. Mamba-based state space modeling is integrated into MAAT to enhance anomaly localization and temporal representation. DiffusionAE learns the manifold of normal signals for reconstruction-based anomaly detection by combining an autoencoder structure with a denoising diffusion probabilistic model. The GDN provides a structure-aware method that is ideal for high-dimensional correlated data by modeling MTS as graph structures and identifying anomalies by learning to measure deviations from graph-based normal patterns.

### 6.4. Evaluation Metrics

This work employs two robust metrics recently proposed for time series anomaly detection to assess performance: an area under the curve (AUC) of the F1-score at *K* different levels (F1*_K_*-AUC), and an AUC of receiver operating characteristic (ROC) at *K* different levels (ROC*_K_*-AUC) [21]. Prior methods [42,43] often overestimate performance by considering a segment as detected if even a single anomalous point is correctly predicted. In contrast, F1*_K_*-AUC is obtained by computing the F1-score across $K\in \left\{0,1,2,\ldots ,W\right\}$ and calculating the area under the curve, where a segment is detected only if at least *K*% of its anomalous points are identified. This work calculates the anomaly score threshold *δ* across 50 threshold values $\delta \in \left\{\frac{k}{49}\cdot s_{max}|k=0,1,\ldots ,49\right\}$, where $s_{max}$ denotes the maximum anomaly score across all traces in the validation set. The threshold *δ* that results in the highest F1*_K_*-AUC in the validation set is applied for an evaluation based on the test set. This work also reports ROC*_K_*-AUC to remove the reliance on a threshold *δ*. ROC*_K_*-AUC is calculated by measuring the true positive rates and false positive rates across thresholds *δ* and *K* values. A threshold-independent comparison of various models is possible by reporting the resulting area under this 2D surface as ROC*_K_*-AUC.

## 7. Performance Evaluation

This section presents the quantitative results from the evaluation, highlighting how GraphDiffusion performs against traditional machine learning, convolutional autoencoders, and transformer- and diffusion-based models. The ablation study evaluates the contribution of the graph topology selection and sliding window parameters. This result demonstrates the superiority of the proposed framework in capturing spatial and temporal patterns, which are critical for DAS anomaly detection.

### 7.1. Comparison with Conventional Anomaly-Detection Models

This work compares the suggested GraphDiffusion with a wide range of generative anomaly-detection models, including diffusion-based generative models, convolutional neural networks, transformer-based architectures, and conventional machine learning methods, to assess its effectiveness. Two evaluation metrics, F1*_K_*-AUC and ROC*_K_*-AUC, evaluate the robustness of anomaly detection under various thresholds and adjustment conditions. The real-time factor (RTF) is also reported to evaluate computational efficiency concerning real-time data rates.

Table 3 summarizes the performance of all comparative models. With an F1*_K_*-AUC of 70.2%, ROC*_K_*-AUC of 73.8%, and RTF of 0.307, OCSVM performs moderately well. Its comparatively poor performance in this DAS raises questions about its ability to model the intricate temporal and spatial dependencies in distributed sensing signals accurately. The CNN-based autoencoder reports an RTF of 0.171, an ROC*_K_*-AUC of 62.7%, and an F1*_K_*-AUC of 79.3%. Its comparatively poor performance might be due to the difficulty in handling distributed or spatially correlated anomalies across DAS channels, although it performs well in reconstructing temporal patterns.

Graph-based deep learning techniques yield measurable improvements. Using spatiotemporal GNNs, GDN maintains an RTF of 0.776 while producing an F1*_K_*-AUC of 86.9% and an ROC*_K_*-AUC of 73.9%. The GDN achieves the second-best performance after the proposed GraphDiffusion highlighting the strength of GNNs in capturing learning spatial and temporal representations in DAS. Transformer-based models yield different outcomes. With a ROC*_K_*-AUC of 68.3% and an F1*_K_*-AUC of 77.8%, TranAD leads this category, outperforming iTransformer, MAAT, and Anomaly Transformer. However, all these transformer variations still lag diffusion-driven and graph-based approaches, presumably due to their limitations in generalizing to anomalies that are spatially distributed or low signal-to-noise conditions, which are typical of DAS.

With an F1*_K_*-AUC of 82.0% and an ROC*_K_*-AUC of 70.7%, DiffusionAE, which combines a DDPM with an autoencoder backbone, outperforms all transformer-based models in terms of detection capability. This finding illustrates how denoising-based generative modeling can be applied to detect anomalous deviations and capture the temporal distribution of normal signals. However, among all the evaluated models, its high RTF of 1.382 makes it the least effective.

With an F1*_K_*-AUC of 98.2%, ROC*_K_*-AUC of 98.0%, and an RTF of 0.890, the proposed GraphDiffusion model provides best performance. The GraphDiffusion achieves a 35.6% lower RTF and improves the F1*_K_*-AUC and ROC*_K_*-AUC by 16.2% and 27.3%, respectively, in comparison to the DiffusionAE. The significant performance margin emphasizes the importance of including the spatial structure in temporal generative models. In contrast to DiffusionAE, GraphDiffusion applies a graph structure to encode the physical layout of DAS channels and employs GCNs to learn spatial dependencies. The proposed model can identify subtle and spatially distributed anomalies that are often ignored by models processing time series separately because it combines diffusion-based temporal modeling with spatially aware representation learning.

### 7.2. Effect of the Graph Topology and Distance Threshold in the Proposed GraphDiffusion

We carried out an ablation study across several configurations to evaluate the influence of the graph topology and spatial distance threshold, *d*, in edge construction based on the performance of the GraphDiffusion. Table 4 summarizes the results of the ablation studies measuring the performance under different thresholds and graph topologies. We evaluated two graph construction strategies with different values of d: an index-based topology (called “normal”) and a 2D coordinate-based topology (called the “two-row topology”).

If the absolute difference between the indices of the two channels is less than or equal to the threshold, *d*, then an edge is created between them in the index-based topology. As shown in Table 4, both F1*_K_*-AUC and ROC*_K_*-AUC increase as *d* rises from 1 to 3, peaking at 97.2% and 89.8%, respectively, at *d* = 3. However, F1*_K_*-AUC slightly decreases to 96.8% at *d* = 4 and then decreases to *d* = 5, indicating that oversmoothing may result from excessive neighborhood aggregation.

Channels in the two-row topology are symmetrically placed along the top and bottom fence edges, resembling the physical configuration of actual DAS installations in a 2D coordinate system. By defining the edges according to the Euclidean distance between channel coordinates, connectivity is possible horizontally, vertically, and diagonally. Oversmoothing occurs at lower *d* values than in the index-based topology because the 2D adjacency, which includes vertical and diagonal connections, allows even a small distance threshold (e.g., *d* = 3) to encompass a larger set of neighbors. As shown in Table 4, the two-row topology produces the best overall results with an F1*_K_*-AUC of 98.2% and an ROC*_K_*-AUC of 98.0% at *d* = 1.5.

Additionally, this work includes a DDPM baseline devoid of any graph-based spatial modeling to separate the contribution of the GNN. The performance of this configuration was lower than the GraphDiffusion, achieving an F1*_K_*-AUC of 81.6% and ROC*_K_*-AUC of 68.8%.

These findings reveal that anomaly-detection performance is significantly influenced by the graph topology design and distance threshold *d*. In addition, by incorporating the spatial structure through the graph topology based on physical information and GNN, more precise modeling of the acoustic signals propagating in DAS systems is possible.

### 7.3. Effect of Window Size in the Proposed GraphDiffusion

We experimented with different window sizes while maintaining a stride of 50% of the window length to assess how the window size affects anomaly-detection performance. This 50% overlap is a commonly used technique in time series anomaly detection, as it ensures that potentially significant events close to window boundaries are not ignored while balancing the computational cost and information preservation. The technique maintains a manageable number of redundant data while more reliably capturing continuous or slowly evolving anomalies by halving the overlap between consecutive windows.

Table 5 summarizes the resulting performance metrics, which explain how various window sizes influence detection accuracy. The performance results indicate a steady improvement in detection performance with an increasing window size. Due to the limited temporal context, 50 small windows with a stride of 25 (50% overlapping) exhibit moderate performance at an F1*_K_*-AUC of 93.8% and ROC*_K_*-AUC of 68.9%. When the window size was increased to 100 with the same 50% strides, the performance significantly improved at an F1*_K_*-AUC of 93.9% and ROC*_K_*-AUC of 81.9%. The benefit of a broader temporal view is demonstrated by the notable gains obtained by further increasing the window size to 200, reaching 96.5% for F1*_K_*-AUC and 96.1% for ROC*_K_*-AUC. A window size of 300 and a stride of 150 yield the best results out of all configurations, at an F1*_K_*-AUC of 98.2% and an ROC*_K_*-AUC of 98.0%. These results highlight that a window size of 300 with a 50% stride achieves optimal detection accuracy in DAS anomaly-detection tasks while maintaining efficiency.

### 7.4. Hidden-Layer-Size Effects in the GNN of GraphDiffusion

We performed an ablation study in GraphDiffusion, fixing the window size to 300 and varying the size of the hidden layer in the GNN. We evaluated four configurations with sizes of 100, 150, 300, and 600 for the hidden layer and reported the performance and model size. Table 6 summarizes these outcomes. Moving from 100 to 150 slightly increases the trainable parameters from 9.95 M to 9.98 M but delivers a considerable increase in performance, with the F1*_K_*-AUC rising from 89.3 to 98.2 and ROC*_K_*-AUC increasing from 89.9 to 98.0. Increasing the hidden size from 150 to 300 raises the parameters to 10.07 M, yielding only a modest F1*_K_*-AUC gain from 98.2 to 98.9, whereas the ROC*_K_*-AUC decreases from 98.0 to 93.3. Increasing it to 600 raises the parameters to 10.25 M and reduces the AUC for F1*_K_* and ROC*_K_* to 97.3 and 92.0, respectively.

The model with a hidden-layer size of 100 underfits the data and cannot completely capture the spatial dependencies. In contrast, the larger hidden-layer sizes introduce extra capacity that increases the computational cost without consistent benefits and can reduce robustness, likely due to oversmoothing or overfitting. The hidden-layer size of the 150 configuration offers the best balance, retaining a high F1*_K_*-AUC and ROC*_K_*-AUC value. Based on this evidence, we adopted 150 as the default hidden-layer size to balance performance and stability.

### 7.5. Performance Comparison of the GraphDiffusion and DDPM Based on a Different DAS Dataset

**Figure 6:**
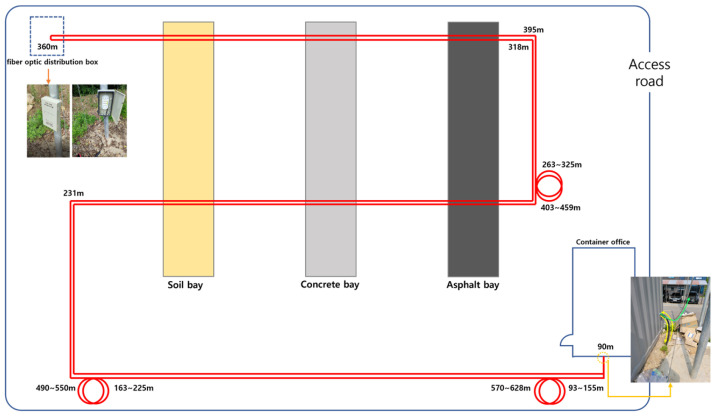
Experimental environment (testbed illustration) where the fiber-optic cable is buried underground.

We employed a different DAS dataset to assess various site configurations and installations, fiber-optic cable layouts, and noise environments. Figure 6 presents the layout of the testbed where the new data collection was conducted. The testbed includes fiber-optic cables laid in a squared S-shaped loop, with three strategically placed stimulation zones: a soil bay, concrete bay, and asphalt bay. The DAS interrogators and patch panels were installed in waterproof containers at the site entrance, and the fiber-optic cables were buried about 50 cm to 1 m underground. Along the buried section, the soil bay experiments included compaction, blasting, and scaffolding effects. The concrete bay and asphalt bay experiments included basket effects, surface scraping, and hydraulic breaker impacts on the concrete and asphalt. The DAS data collection testbed comprises 970 sensors, and the number of DAS data channels is 970.

Using the DAS data, we constructed a training set with 357,100 traces, a validation set with 19,800 normal traces and 20,100 anomalous traces, and a test set with 19,900 normal traces and 20,100 anomalous traces. The window size was set to 100, and the stride size was set to 50 to accommodate the high channel count. After training GraphDiffusion and the general DDPM using the training and validation datasets, performance was measured using the test dataset. The GraphDiffusion model generated a graph topology that mimics the layout of a fiber-optic cable, and the spatial distance threshold was set to *d* = 1.5.

Table 7 contrasts the GraphDiffusion model with a general DDPM. The GraphDiffusion model delivers substantially higher F1*_K_*-AUC and ROC*_K_*-AUC scores compared to the baseline DDPM. This performance improvement underscores the benefit of encoding the fiber-optic cable layout via the GNN, leading to more accurate anomaly detection across diverse DAS data. Although the absolute score based on the new dataset decreased due to the increased layout complexity, underground burial, and anomalous events in various bays, GraphDiffusion still outperformed the baseline DDPM. These results demonstrate that encoding the physical cable layouts using GNNs yields better performance regardless of the installation and noise.

### 7.6. GNN Embedding Analysis Using DAS Signals

**Figure 7:**
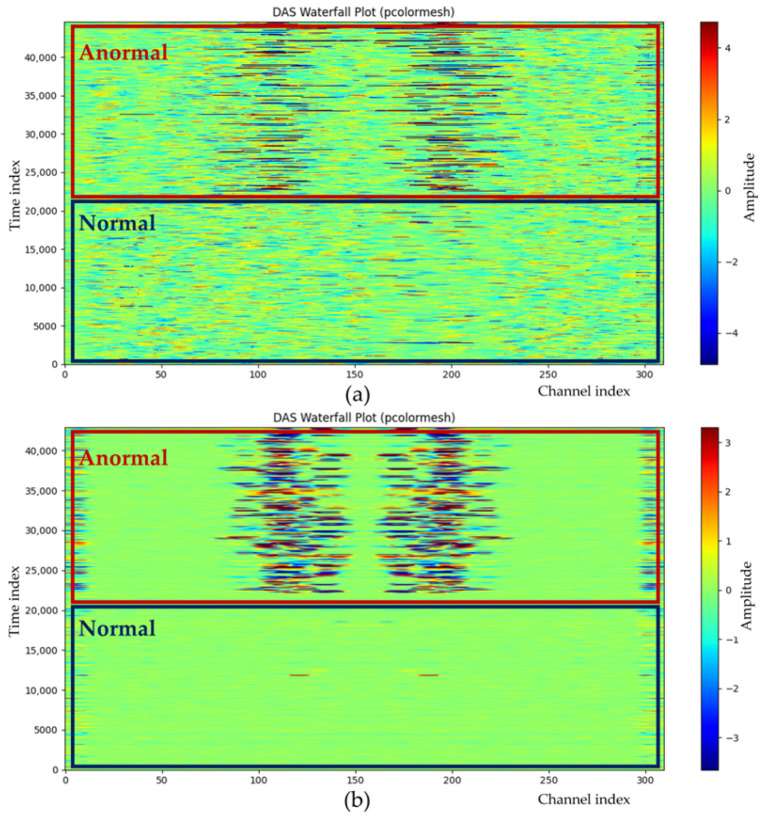
Waterfall plots of (**a**) the original DAS signal and (**b**) the corresponding GNN output.

Spatially structured anomaly patterns highlight the need to model intersensor relationships in DAS anomaly detection. Because acoustic energy propagates along the cable, anomalous signals rarely remain isolated and instead affect adjacent channels. The proposed approach captures these dependencies with a GNN that encodes the DAS sensor layout as a graph and learns spatially coherent features by aggregating information from neighboring sensors via adjacency-based message passing. Figure 7 compares the raw DAS input (a) with the GNN output (b) for the same segment, with sensors on the *x*-axis and time on the *y*-axis. In normal intervals, scattered noise in the input is suppressed, producing a cleaner background in the GNN output. In anormal intervals, banded patterns spanning the neighboring sensors become clearer and more continuous after the GNN. These sharper spatial features are provided to the conditional DDPM, enabling temporal denoising with explicit spatial context and clarifying that the observed gains stem from the GNN-derived spatial abstraction rather than from a plain DDPM.

### 7.7. Discussion

The outcomes of the experiment demonstrate how well the suggested GraphDiffusion works for generative anomaly detection in DAS data. The proposed method overcomes the primary limitations of earlier approaches by combining temporal generative modeling via a conditional DDPM with spatial modeling via a GNN. GraphDiffusion continuously outperforms comparative models, such as GDN, TranAD, and DiffusionAE, in terms of the F1*_K_*-AUC and ROC*_K_*-AUC metrics. This enhancement highlights how crucial it is for DAS to capture intricate temporal dynamics and interchannel dependencies simultaneously.

Additionally, the ablation study reveals that model performance is strongly affected by the graph topology selection. Although index-based adjacent graphs perform well, using graphs with a two-row topology yields even more benefits. This topology improves the ability of the model to represent acoustic propagation across the sensor array by constructing edges according to Euclidean distances. Thus, the model can capture richer spatial relationships, including horizontal, vertical, and diagonal connectivity. The findings imply that a spatially aware graph structure that more accurately reflects the actual arrangement of sensors is advantageous for real-world DAS configurations.

Furthermore, the analysis of the distance threshold, *d*, indicates a nontrivial trade-off. Excessively dense graphs can weaken local structural cues and increase the computational load, but larger values of *d* boost connectivity and might provide each node with more context. A moderate threshold (e.g., *d* = 1.5 in the two-row topology-based graph) strikes the best balance in the experiments, producing the highest ROC*_K_*-AUC and F1*_K_*-AUC scores.

The comparison between diffusion and GraphDiffusion further highlights the contribution of spatial awareness. Diffusion has limited ability to detect anomalies that are scattered or propagated due to a lack of information regarding the spatial structure. GraphDiffusion can contextualize the signal of each channel within its neighborhood by incorporating GNN-based encoding, improving anomaly localization and robustness in complex or noisy environments.

## 8. Conclusions

This study presents GraphDiffusion, a novel method combining the conditional DDPM and GNN for generative anomaly detection in DAS data. The proposed method overcomes the primary limitations of earlier approaches that either ignore the spatial structure or rely heavily on labeled data by modeling the spatial layout of DAS channels as a graph and learning the temporal dynamics via a diffusion-based generative process.

This work demonstrates that a two-row topology-based graph, representing physical relationships, such as horizontal, vertical, and diagonal proximity, significantly improves performance. Experimental results show that the proposed GraphDiffusion achieved the highest F1*_K_*-AUC and ROC*_K_*-AUC scores, corresponding to 98.2% and 98.0%, respectively, outperforming the comparative models.

Additionally, the ablation study reveals that practical trade-offs between locality and connectivity are possible when the spatial distance threshold is tuned during edge construction. The two-row topology achieved optimal performance when the spatial distance threshold *d* was set to 1.5. Reducing *d* from 1.5 to 1 resulted in a 5.2%p drop in the F1*_K_*-AUC and a 14.4%p drop in the ROC*_K_*-AUC, indicating insufficient connectivity. Conversely, increasing *d* to 3 resulted in a marginal 0.1%p increase in the F1*_K_*-AUC but a 7.1%p decrease in the ROC*_K_*-AUC, suggesting that excessive neighborhood aggregation may lead to oversmoothing.

Furthermore, an ablation study that removed the GNN component from the proposed GraphDiffusion revealed that the DDPM without spatial modeling significantly degraded the F1*_K_*-AUC and ROC*_K_*-AUC scores by 16.6%p and 29.2%p, respectively. This finding highlights the crucial role of spatial modeling in capturing interchannel dependencies of anomalies in DAS signals.

However, this study has two limitations. First, since this model relies on a pre-determined graph structure based on the physical layout of the sensors, it may be difficult to generalize deployments with irregular or unknown topologies. In addition, the computational overhead caused by the repeated noise-removal steps in the diffusion model makes it difficult to deploy it in real time.

In future work, we aim to explore an adaptive graph-construction method that can dynamically reflect spatial relationships in the data itself and a lightweight diffusion transformation method to reduce the inference latency and improve the scalability of real-world monitoring systems.

## Figures and Tables

**Table 1:** Operational, experimental parameters for the distributed acoustic sensing system.

Parameter Item	Value	Unit
Spatial resolution	5.1	m
Pulse width	50	ns
Sampling rate	100	Msps
Spatial sampling interval	1.027	m
Pulse repetition rate	20	kHz
Frequency response	DC to 10	kHz

**Table 2:** Data collection for each event scenario.

Event Scenario	Data Points (Traces)
Digging event	347,000
Fence impact (fixed point)	434,100
Fence shaking (fixed point)	528,900
Fence impact (multiple points)	529,300
Ladder intrusion	785,000
Walking (normal)	2,186,200
Running (normal)	1,114,200
Walking (1 m distance)	340,900
Walking (2 m distance)	348,700
Walking (3 m distance)	174,500
Walking (4 m distance)	166,900
Walking (5 m distance)	186,400

**Table 3:** Performance comparison of the conventional and proposed anomaly-detection models measured based on an area under the curve (AUC) of the F1-score at *K* different levels (F1*_K_*-AUC), an AUC of receiver operating characteristic (ROC) at *K* different levels (ROC*_K_*-AUC), and the real-time factor (RTF).

Model	F1*_K_*-AUC	ROC*_K_*-AUC	RTF
Autoencoder [9]	79.3	62.7	0.171
OCSVM [41]	70.2	73.8	0.307
GDN [32]	86.9	73.9	0.776
AnomalyTransformer [28]	77.0	68.1	0.192
TranAD [29]	77.8	68.3	0.220
iTransformer [30]	71.3	62.0	0.164
MAAT [31]	73.0	66.8	0.906
DiffusionAE [21]	82.0	70.7	1.382
GraphDiffusion (proposed)	98.2	98.0	0.890

**Table 4:** Ablation study on the graph topology and spatial distance threshold (*d*) in GraphDiffusion.

Architecture	Graph Topology	*d*	F1*_K_*-AUC	ROC*_K_*-AUC
GraphDiffusion	Normal	1	92.7	80.5
		2	94.4	85.8
		3	97.2	89.8
		4	96.8	93.0
		5	94.6	83.4
	Two-row topology	1	93.0	83.6
		1.5	98.2	98.0
		3	98.3	90.9
DDPM	No GNN	-	81.6	68.8

**Table 5:** Anomaly detection for varying window sizes with a 50% stride.

Window Size	F1*_K_*-AUC	ROC*_K_*-AUC
50	93.8	68.9
100	93.9	81.9
200	96.5	96.1
300	98.2	98.0

**Table 6:** Anomaly detection for varying the sizes of the hidden layers.

Hidden Layers	Parameters	F1*_K_*-AUC	ROC*_K_*-AUC
100	9.95 M	89.3	89.9
150	9.98 M	98.2	98.0
300	10.07 M	98.9	93.3
600	10.25 M	97.3	92.0

**Table 7:** Performance comparison of the GraphDiffusion and DDPM based on DAS data with different site configurations, fiber-optic cable layouts, and noise environments.

Architecture	*d*	F1*_K_*-AUC	ROC*_K_*-AUC
DDPM	-	75.0	69.2
GraphDiffusion	1.5	88.1	89.6

## Data Availability

The data used to develop this system were obtained within the framework of the project. The data are currently unavailable online. For more information on the data, please contact the authors.
